# Editorial: Nanotechnology-based detection, prevention and treatment of infectious diseases

**DOI:** 10.3389/fmicb.2023.1224727

**Published:** 2023-06-01

**Authors:** Abbas Hajizade, Gholamreza Ahmadian, Kelong Fan, Mingzhao Zhu, Ayyoob Arpanaei

**Affiliations:** ^1^Biology Research Center, Faculty of Basic Sciences, Imam Hossein University, Tehran, Iran; ^2^Department of Industrial and Environmental Biotechnology, National Institute for Genetic Engineering and Biotechnology, Tehran, Iran; ^3^CAS Engineering Laboratory for Nanozyme, Institute of Biophysics, Chinese Academy of Sciences, Beijing, China; ^4^Key Laboratory of Infection and Immunity, Institute of Biophysics, Chinese Academy of Sciences, Beijing, China; ^5^Scion, Rotorua, New Zealand

**Keywords:** nanomaterials, nano-vaccines, nano-biosensors, nano-delivery systems, microbes, infectious diseases

Infectious diseases are a significant global concern caused by bacterial, viral, fungal, and parasitic agents, with varying levels of severity. Timely detection, prevention, and treatment of these agents can dramatically save lives and costs. The emergence of SARS-CoV-2, the causative agent of the coronavirus disease 2019 (COVID-19), has prompted the healthcare industry to develop new, effective strategies for managing infectious diseases.

Nanotechnology is an emerging tool that has revolutionized various aspects of human life, including healthcare, agriculture, cosmetics, electronics, etc. In medicine and healthcare fields, nanotechnology has given tremendous effects from basic sciences to clinical settings. This approach has reformed the detection methods, prevention strategies, and treatment of different diseases, including infections and has helped to understand the mechanisms of different diseases better.

There are several commercially available nano-based detection kits for the diagnosis of microbial pathogens. Prevention of infectious diseases via vaccination is another field in which nanotechnology has proven its outstanding capabilities. It has been shown that many nanomaterials are suitable carriers for the delivery of different vaccines. Furthermore, many nanomaterials have intrinsic adjuvanticity properties, which makes them appropriate tools for the delivery of vaccines. The delivery of antimicrobial agents using nanomaterials is also a growing field that improves the efficiency of antimicrobials activity and reduces the side effects of these compounds.

This topic aims to uncover the role of nanotechnology in combating infectious agents. Six articles have been accepted and published in this field. The finding of these articles showed the remarkable potential of nanomaterials and nanotechnology-based techniques in the development of detection methods, prevention strategies, and treatment of infectious diseases.

Yang, Wang et al. conducted a study where they designed, fabricated, and tested a multiple cross displacement amplification (MCDA) technique combined with a gold nanoparticle (NP)-based lateral flow assay for the detection of *Brucella abortus* (*B*. *abortus*) in the blood samples. The limit of detection (LoD) of their developed method was calculated as 10 fg/μL (~3 copy/μL). The developed method showed high specificity for detecting *B. abortus* and overcame the limitations of previous detection methods. The new system allowed for easy, rapid, reliable, and cost-effective detection of the bacterium.

Yang, Huang et al. also demonstrated the potential of a nano-based system in the detection and differentiation of bacterial pathogens in another research. They developed a novel nano-based diagnostic method for the rapid detection of *Mycobacterium tuberculosis* (MTB), the causative agent of tuberculosis, and its differentiation from other *Mycobacterium tuberculosis* complex (MTBC) members, e.g., *M. bovis, Mycobacterium microti, Mycobacterium africanum*. This system employs multiplex loop-mediated isothermal amplification (mLAMP) combined with a NP-based lateral flow biosensor (LFB) (mLAMP-LFB). The LoD of the system was 125 fg per vessel for the pure genomic DNA of MTB and 4.8 × 103 CFU/mL for the sputum samples. The specificity of the system was 100%. The entire detection process could be completed in about 80 min. Based on these results, they concluded that mLAMP-LFB is a rapid, reliable, and sensitive method that is able to detect representative members of MTBC and simultaneously differentiate MTB from other MTBC members. It has the potential to be used as a screening tool for tuberculosis in clinical, field, and basic laboratory settings.

Shen et al. synthesized gold nanoclusters (AuNCs) that were functionalized with an antibacterial peptide (CWR11: CWFWKWWRRRRR). The exploited AuNCs had fluorescence activity. They found that the conjugation of the peptide to the NCs retained the bacteriostatic and bacteriocidal activity of the peptide. Indeed, the cytotoxicity of the peptide following the conjugation to the AuNCs was significantly decreased. Furthermore, the AuNCs had the ability to bind to the bacterial cells, hence, it is a suitable nanodevice for *in vivo* imaging. This study highlights the potential of nanomaterials for developing biocompatible antibacterial substances and their suitability for *in vivo* imaging.

*Toxoplasma gondii* (*T. gondii*), the etiological agent of toxoplasmosis, is an intracellular protozoan that affects about 30–50% of the world's population and is a main public health problem. Congenital toxoplasmosis, in the first gestational months leads to miscarriage, stillbirth, premature birth, malformations, and neurological and/or ocular disorders in newborns infected by *T. gondii*. Conventionally, congenital toxoplasmosis has been treated by the combination of sulfadiazine, pyrimethamine and folinic acid. However, it has many side effects, such as bone marrow suppression and teratogenic effects. By using biogenic silver NPs (AgNp-Bio), Costa et al. developed a new therapeutic strategy to reduce these effects and improve the control of the infection. They investigated the effects of AgNp-Bio on BeWo and HTR-8/SVneo cells, and villous explants and its effects against *T. gondii* infection. They found that AgNp-Bio can decrease *T. gondii* infection in trophoblast cells and villous explants through the induction of inflammatory mediators in the cells and independent of mediators in the chorionic villus. Based on these results, they concluded that AgNP-Bio-based treatment is a promising strategy for combating *T. gondii* infection.

Zong et al. conducted a study to investigate the antibacterial and biofilm performance of quaternized chitosan (QCh) modified upconversion nanoparticles (UCNP)@SiO2/methylene blue (MB) against *Enterococcus faecalis* (*E. faecalis*) in apical root canals. *E. faecalis* can form biofilm in root canals and cause persistent endodontic infections (PEIs). Nowadays, canal disinfectants such as calcium hydroxide and chlorhexidine are used to overcome this problem; however, these disinfectants are not efficient enough to eradicate bacteria and/or biofilms. Antimicrobial photodynamic therapy (aPDT) has had a promising effect on the eradication of bacteria and biofilms. Following the preparation of UCNP@SiO2/MB, they were coated with QCh. This coating increased the conversion efficiency of UCNP to generate more reactive oxygen species (ROS). Furthermore, the prepared UCNP@SiO2/MB@QCh exhibited highly effective antibacterial activity against free *E. faecalis* and related biofilm *in vitro* and extracted teeth. *In vitro* cellular tests also showed that UCNP@SiO2/MB@QCh had acceptable cytocompatibility. Therefore, UCNP@SiO2/MB@QCh could offer a novel strategy for potential clinical applications of aPDT in the treatment of PEIs.

In a great review paper, Ren et al. comprehensively studied the recent findings regarding the role of functionalized NPs in the prevention and targeted therapy of viruses that have neurotropism properties, including herpes simplex virus (HSV), Zika virus (ZIKV), rabies virus (RABV), and severe acute respiratory syndrome coronavirus type 2 (SARS-CoV-2). They reviewed and discussed the performance of the peptide-, antibody-, receptor-, and antigen-functionalized NPs in the prevention and therapy of viral infections. By reviewing the latest and most important studies in this field, they provided a good reference for improving our understanding of the potential applications and future of functionalized NPs in the prevention and treatment of diseases caused by viruses with neurotropism properties.

In conclusion, nanomaterials and nanotechnology-based methods are increasingly used for the management of the infectious diseases. Combined with other approaches, such as molecular techniques and serological methods, nanotechnology-based tools can be used as sensitive and specific diagnostic systems; they can act as effective carriers for the delivery of vaccines and exert their roles in preventing the diseases, especially infectious ones. Furthermore, some nanomaterials have intrinsic antimicrobial properties and at the same time they can be exploited as a delivery system for therapeutic agents and hence increase the efficiency of the treatment regimens. Undoubtedly, further opportunities exist and need to be explored to develop innovative nanotechnology-based approaches for more efficient diagnosis and treatment of various diseases.

## Author contributions

All authors listed have made a substantial, direct, and intellectual contribution to the work and approved it for publication.

